# Persistent left ventricular dysfunction after acute lymphocytic myocarditis: Frequency and predictors

**DOI:** 10.1371/journal.pone.0214616

**Published:** 2019-03-28

**Authors:** Marco Merlo, Enrico Ammirati, Piero Gentile, Jessica Artico, Antonio Cannatà, Gherardo Finocchiaro, Giulia Barbati, Paola Sormani, Marisa Varrenti, Andrea Perkan, Enrico Fabris, Aneta Aleksova, Rossana Bussani, Duccio Petrella, Manlio Cipriani, Claudia Raineri, Maria Frigerio, Gianfranco Sinagra

**Affiliations:** 1 Cardiovascular Department, Azienda Sanitaria Universitaria Integrata (ASUITS) and University of Trieste, Trieste, Italy; 2 “De Gasperis” Cardio Center, Niguarda Hospital, Milan, Italy; 3 Cardiology Clinical and Academic Group, St. George’s, University of London, London, United Kingdom; 4 Biostatistics Unit, Department of Medical Sciences, University of Trieste, Trieste, Italy; 5 Department of Pathology, Azienda Sanitaria Universitaria Integrata (ASUITS) and University of Trieste, Trieste, Italy; 6 Pathology Laboratories, Niguarda Hospital, Milan, Italy; 7 Department of Cardiology, Fondazione IRCCS Policlinico S.Matteo, Pavia, Italy; Scuola Superiore Sant’Anna, ITALY

## Abstract

**Background:**

Persistent left ventricular (LV) systolic dysfunction in patients with acute lymphocytic myocarditis (LM) is widely unexplored.

**Objectives:**

To assess the frequency and predictors of persistent LV dysfunction in patients with LM and reduced LVEF at admission.

**Methods and results:**

We retrospectively evaluated 89 consecutive patients with histologically-proven acute myocarditis enrolled at three Italian referral hospitals. A subgroup of 48 patients with LM, baseline systolic impairment and an available echocardiographic assessment at 12 months (6–18) from discharge constituted the study population. The primary study end-point was persistent LV dysfunction, defined as LVEF <50% at 1-year, and was observed in 27/48 patients (56.3%). Higher LV end-diastolic diameter at admission (odds ratio [OR] 1.22, 95% confidence interval [CI] 1.04–1.43, p = 0.002), non-fulminant presentation (OR 8.46, 95% CI 1.28–55.75, p = 0.013) and presence of a poor lymphocytic infiltrate (OR 12.40, 95% CI 1.23–124.97, p = 0.010) emerged as independent predictors of persistent LV dysfunction at multivariate analysis (area under the curve 0.91, 95% CI 0.82–0.99). Pre-discharge LVEF was lower in patients with persistent LV dysfunction compared to the others (32%±8 vs. 53%±8, p <0.001), and this single variable showed the best accuracy in predicting the study end-point (area under the curve 0.95, 95% CI 0.89–1.00).

**Conclusions:**

More than half of patients presenting with acute LM and LVEF <50% who survive the acute phase show persistent LV dysfunction after 1-year from hospital discharge. Features of subacute inflammatory process and of established myocardial damage at initial hospitalization emerged as predictors of this end-point.

## Introduction

Acute lymphocytic myocarditis (LM) presenting with left ventricular (LV) systolic dysfunction represents a challenge in terms of diagnosis, management and prognostication [1–5]. Previous studies showed that LV systolic dysfunction predicts poor in-hospital and long-term prognosis in histologically-proven acute myocarditis [1, 2, 6] Moreover, patients with acute LM with fulminant presentation, characterized by hemodynamic instability [7], have a worse in-hospital prognosis and are more prone to exhibit LV systolic dysfunction during follow-up with respect to those with non-fulminant presentation [1, 8]. However, the natural history of the specific subgroup of patients presenting with acute LM and LV systolic dysfunction is widely unknown, particularly regarding the frequency and the early predictors of persistent LV dysfunction in the long term. These issues are relevant for defining tailored follow-up and therapy in these high-risk patients. The aims of this study were: 1) to assess the proportion of patients that will show persistently reduced LV ejection fraction (LVEF) at 1 year among those with histologically-proven acute LM and impaired LV systolic function ad admission, and 2) to identify early predictors of persistent LV systolic dysfunction.

## Methods

### Study population

**Fig 1:**
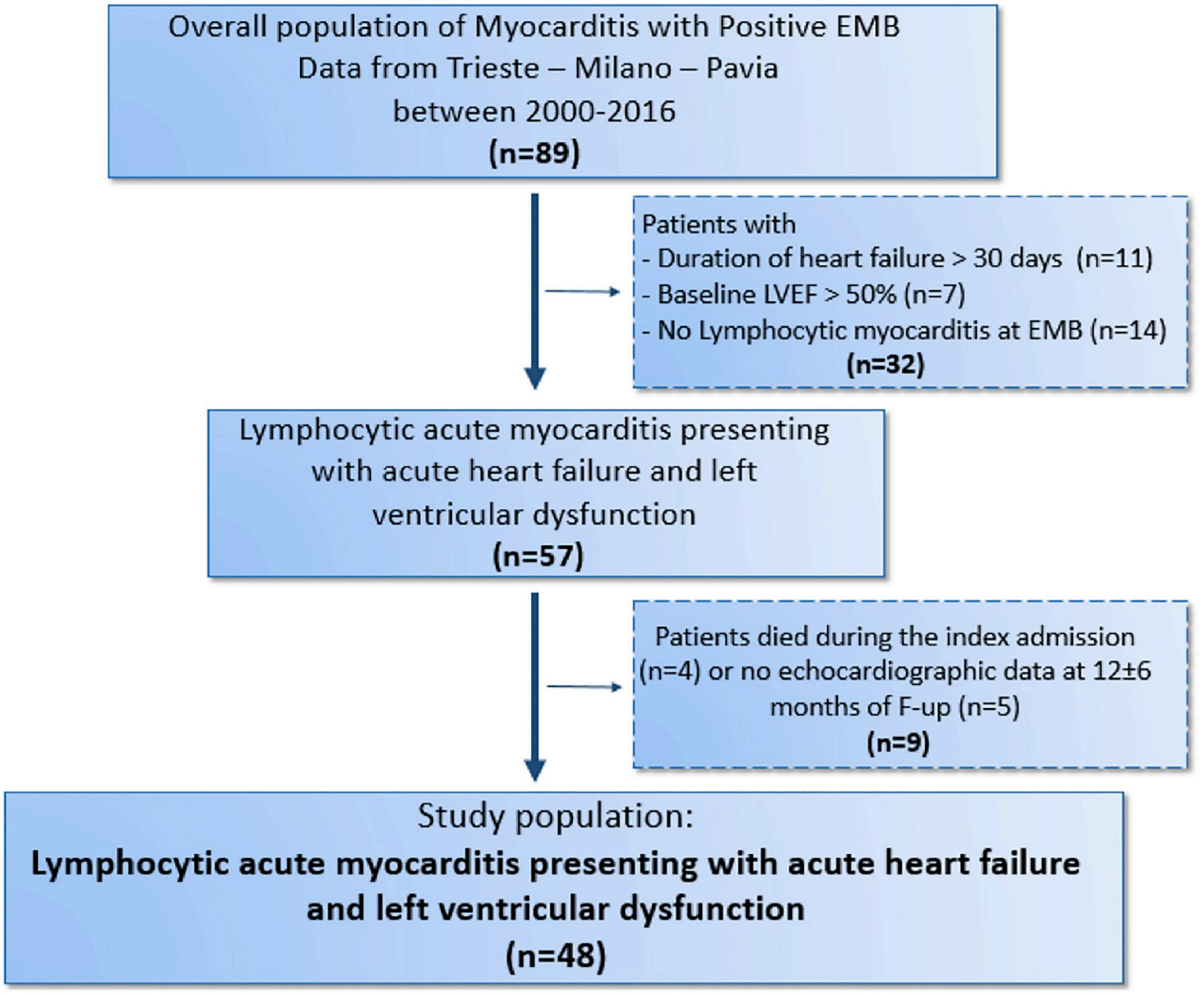
Flow diagram describing the selection of the study population. EMB, Endomyocardial biopsy.

We retrospectively analyzed all the patients with histologically proven acute myocarditis consecutively admitted at 3 Italian referral Centers for cardiomyopathies (Cardiovascular Department of Trieste, De Gasperis Cardio Center, Niguarda Hospital of Milan and San Matteo Hospital of Pavia) from 2000 to 2016. For the purpose of this study, the following inclusion criteria were applied: 1) duration of heart failure (HF) symptoms ≤30 days; 2) impaired LV function (echocardiographic LV ejection fraction, LVEF, <50% at presentation; 3) histology and immunohistochemistry findings consistent with LM at endomyocardial biopsy, according to international criteria [9]; 4) available clinical and echocardiographic assessment within 12 (6–18) months. Among 89 consecutively enrolled patients with biopsy-proven acute myocarditis (46 from the Trieste Cardiovascular Department, 38 from the De Gasperis Cardio Center, Niguarda Hospital, Milan, 5 from the Cardiovascular Department, Policlinico San Matteo, Pavia), 57 (64.0%) patients were classified as LM and had LV systolic dysfunction. Four patients (5.2%) with fulminant myocarditis died or underwent heart transplant during the initial hospitalization. Five (8.7%) patients surviving more than 1 year after hospital discharge had no available follow-up echocardiographic information. Thus, the study population consisted of the remaining 48 patients (Fig 1). The clinical and echocardiographic evaluation for the assessment of possible persistent LV systolic dysfunction was performed at a median time of 10 months (interquartile range, 8 to 12).

The study was approved by the Ethic Committees of Trieste, Milan and Pavia Hospitals. The study complied with the declaration of Helsinki.

### Patients management

All the patients underwent a thorough invasive and non-invasive assessment at index hospitalization. In all the patients surviving the acute phase, clinical follow-up within 12 (6–18) months from index hospitalization was scheduled, including clinical evaluation, laboratory testing, and echocardiographic assessment.

### Endomyocardial biopsy

Endomyocardial biopsy (EMB) was performed from either the left or the right ventricle according to the Center experience or single case evaluation. The mean number of histological samples per patient was 4 (range 1–6) [10, 11]. Samples for histopathological analysis were fixed in 10% buffered formalin, paraffin embedded, sectioned on multiple levels at 2 μm, and stained with Hematoxylin-Eosin (HE), Azan Mallory trichrome and Weigert van Gieson stains. Congo Red staining for amyloid, Perl’s staining for iron deposits and colloidal iron staining for amorphous substance in the young connective tissue was performed if necessary. The following antigens were tested if necessary using specific antibodies for identification of myocardial inflammation and for the identification, localization, and characterization of mononuclear cell infiltrates: human leukocyte antigen (HLA)-DR-α to assess HLA class II expression in antigen-presenting immune cells; CD3 for T cells; CD4 for helper T-cells; CD8 for suppressor T-cells; CD68 KP1 and CD 163 for activated macrophages. In all cases a significant lymphocytic infiltrate was present [12] and was associated with a positive immunohistochemistry analysis. The grading of the myocardial inflammatory infiltrates was performed blindly on a scale of poor, moderate and plentiful. ‘Poor’ represented focal distribution of myocardial lesions with a diameter < 100 μm, ‘plentiful’ indicated the presence of multiple lesions over the entire sample, while ‘moderate’ denoted intermediate severity. The most frequently scored severity of cellular infiltration and myocardial necrosis in each stain was considered representative of myocardial pathology [13]. Samples from patients without an acute fulminant condition were evaluated for the presence of the genome of cardiotropic viruses (Parvovirus B19, Adenovirus, Enterovirus, Ebstein-Barr Virus, Herpes Simplex Virus 1, Herpes Simplex Virus 2) by Real Time Polymerase Chain Reaction (PCR) using specific primers and probes. In case of virus-positive EMB, blood samples were also tested for the same virus. In fulminant forms, viral PCR were not routinely performed. All specimens have been carefully evaluated by the resident pathologist.

### Therapy

When clinical condition was sufficiently stable, patients received recommended HF medical treatment as indicated by current guidelines. Patients with fulminant forms, defined by the need for inotropes or vasopressors and/or mechanical circulatory support during the acute phase [7, 8], were mostly treated with intravenous corticosteroids early after histological confirmation as previously reported [8]. In the other patients, immunosuppressive therapy was administrated in the presence of 1) myocardial immune activation at immunohistochemistry analysis, 2) persistent LV dysfunction under standard treatment[5], and 3) absence of viral genome in myocardial cells by Real Time Polymerase Chain Reaction. Immunosuppressive therapy consisted of prednisone (50 mg/m2/day with progressive downscaling) and azathioprine (75 mg/m2/day) for a 6-month period [1].

### Echocardiographic assessment

Echocardiographic assessment consisted in comprehensive M-mode, 2-dimensional and Doppler studies. Systolic and diastolic ventricular function and valve regurgitations were defined according to international guidelines [14]. In particular, LVEF was calculated from 2-dimensional apical 4 and 2 chambers approach using the biplane method of discs (modified Simpson’s rule). LVEF was systematically measured at admission, prior to discharge and at follow-up in all patients included.

### Study design and end-points

The primary study end-point was the persistence of a LVEF <50% at follow-up [15]. Secondary end-point was the long-term survival-free from cardiovascular death or heart transplant (HTx).

Follow-up information were obtained through phone calls with patients, their relatives, or general practitioner or by consulting the office of national statistics. The end of follow-up was considered as June 30, 2017 (last check date of status for alive patients) or the date of death or HTx.

### Statistical analysis

Summary statistics of clinical and laboratory variables were expressed as mean and standard deviation, median and interquartile range or counts and percentage, as appropriate (Shapiro-Wilk test was used to test for normal distribution of continuous variables). Comparisons between groups were made by the ANOVA test on continuous variables, using the Brown-Forsythe statistic when the assumption of equal variances did not hold, or the non-parametric Mann-Whitney test when necessary. The Chi-square or Fisher’s exact test were calculated for discrete variables. Uni- and multi-variable logistic regression analyses were estimated to determine the most predictive combination of independent factors associated with the primary end-point, by means of a full-model strategy applied each time starting from a different subset of at most three parameters, due to the low event rate. For each combination of parameters, the predictive accuracy of the corresponding vector of estimated probabilities of event was evaluated by means of a receiver operating characteristic (ROC) curve. Since the limited number of events, the De Long test between areas under the curves (AUCs) had a limited power, and for this reason the model with the absolute highest accuracy was retained. This final model was internally validated with a bootstrap-based procedure, in order to account for the optimism in the AUC estimate [16]. To explore the secondary end-point, i.e. the long-term survival outcome, Kaplan-Meier survival curves were estimated (starting from the date of re-evaluation) and the Log-rank test was performed.

Linear association between variables was analysed using Pearson’s correlation coefficient for normally distributed variables and Spearman’s correlation coefficient for not normally distributed variables and significance of both coefficients was reported. The IBM-SPSS version 19 and the R statistical software version 3.4.0, libraries “survival” and “rms” were used for statistical analyses.

## Results

### Study population

Baseline characteristics of the studied cohort (n = 48) are summarized in Table 1 (first column). Mean age was 38±16 years, and 52% were males. The median time between the onset of symptoms and hospitalization 11 days (interquartile range, 5 to 26). Most of the patients (n = 40, 83%) presented a flu-like syndrome before the onset of HF symptoms, but only 24 (50%) showed an increased C-reactive protein at admission. The mean LVEF was 26±9% and the mean LV end-diastolic diameter [EDD] was 57±9 mm. PCR analysis was positive in 8 (24%) out of 33 tested patients, in all cases for Parvovirus B19. Immunosuppressive therapy was administrated in 73% of patients.

**Table 1:** Baseline characteristics of study population divided on the basis of persistent left ventricular (LV) systolic dysfunction.

	Study population	Persistent LV dysfunction at follow-up	Normal LV function at follow-up	p
	(N = 48)	(N = 27, 56%)	(N = 21, 44%)	
Age (years)	38±16	43±14	32±15	0.03
Age <15 (%)	2 (4)	1 (4)	1 (5)	0.856
Male gender, n (%)	25 (52)	14 (52)	11 (52)	0.601
Duration of symptoms (days)	11 (5–26)	20 (14–28)	5 (3–6)	<0.001
Admission Heart Rate (bpm)	95±34	81±25	114±36	0.001
Admission SBP (mmHg)	103±21	110±16	87±23	0.002
NYHA Class				
II	17 (35)	11 (41)	7 (33)	0.880
III	14 (29)	12 (44)	2 (10)	0.008
IV	16 (33)	4 (15)	12 (60)	0.001
Fulminant forms, n (%)	23 (48)	6 (22)	17 (81)	<0.001
Flu-like symptoms, n (%)	40 (83)	20 (74)	20 (95)	0.087
Increased CRP, n (%)	24 (50)	8 (30)	16 (76)	<0.001
Atrial Fibrillation, n (%)	5 (10)	1 (4)	4 (19)	0.09
LBBB, n (%)	3 (6)	1 (4)	2 (10)	0.693
1st, 2nd, 3rd AV Blocks, n (%)	7 (15)	4 (15)	3 (14)	0.623
LVEDD (mm)	57±9	60±6	51±10	<0.001
Baseline LVEF (%)	26±9	28±7	24±11	0.211
LVEF at discharge (%)	42±13	32±8	53±8	<0.001
Pericardial effusion, n (%)	13 (27)	4 (15)	9 (43)	0.039
Poor lymphocytic Infiltrate*, n (%)	15 (31)	13 (48)	2 (10)	0.008
Moderate-to severe fibrosis at EMB	16 (33)	10 (37)	6 (29)	0.550
Beta-blockers at discharge, n (%)	38 (79)	25 (93)	13 (62)	0.012
ACE-inhibitors-ARBs at discharge, n (%)	43 (90)	24 (89)	19 (90)	0.621
Aldosterone receptors antagonist at discharge, n (%)	18 (38)	12 (44)	6 (29)	0.205
Immunosuppressive therapy (%)	35 (73)	20 (74)	15 (71)	0.838

Values are expressed as mean±SD or median with interquartile range as appropriate, and as percentage.

ACE, angiotensin converting enzyme; ARBs, angiotensin receptor blockers; AV, atrioventricular; CRP, C-reactive protein; EMB: endomiocardial biopsy; LBBB, left bundle branch block; LM: lymphocytic myocarditis; LVEDD, left ventricular end-diastolic diameter; LVEDV, left ventricular end-diastolic volume; LVEF, ejection fraction; MR, mitral regurgitation; RFP, restrictive filling pattern; SBP, systolic blood pressure;

*vs. moderate to plentiful

#### Baseline predictors of persistent LVEF<50% at follow-up

**Fig 2:**
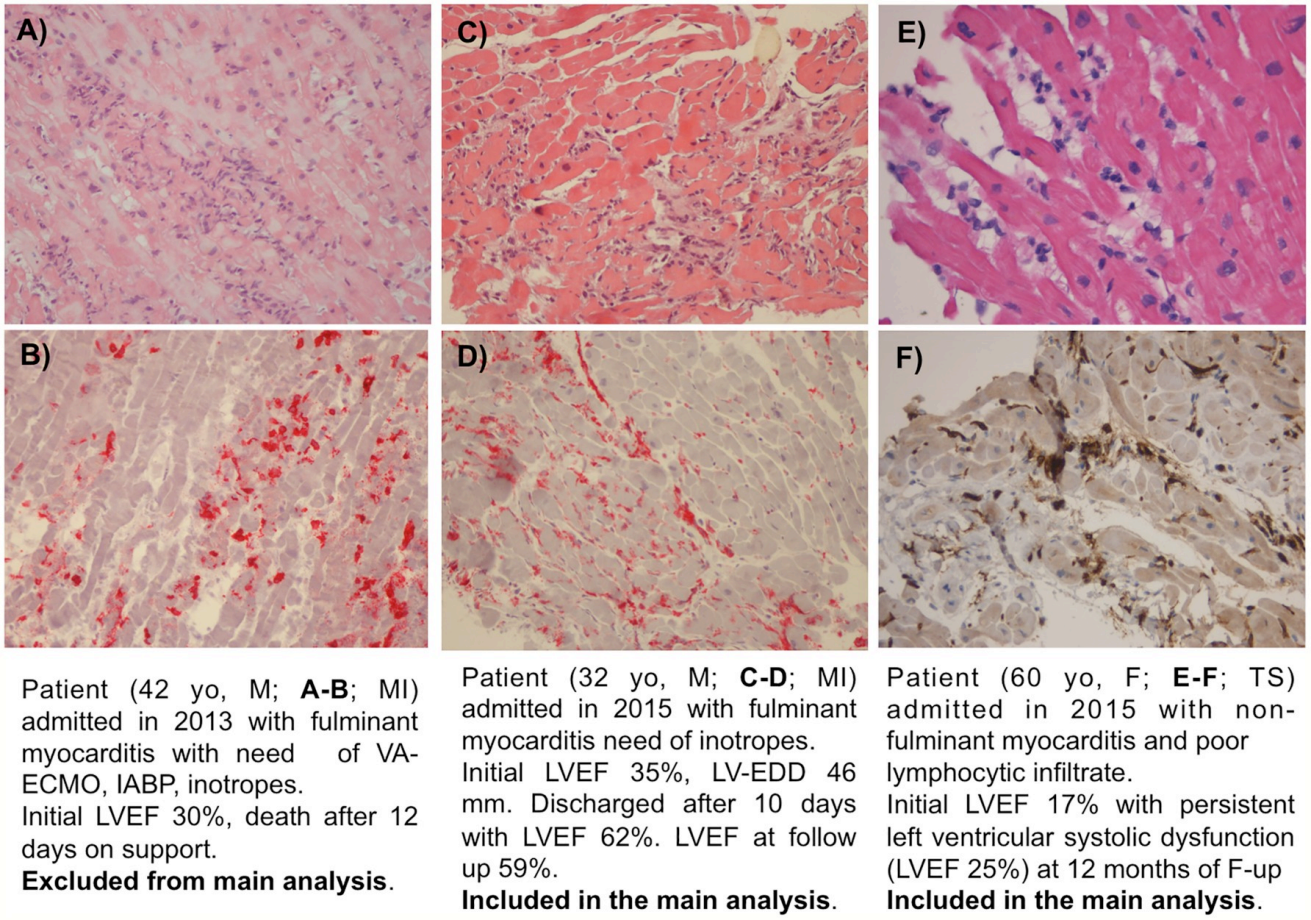
Histological images of three patients with acute LM presenting left ventricular dysfunction at admission. A-B) Fulminant form with plentiful lymphocytic infiltrate and necrosis, he died during the acute phase (excluded from main analysis); C-D) Fulminant form with plentiful lymphocytic infiltrate, he normalized systolic function during follow-up (included in the main analysis); E-F) non-fulminant form with poor lymphocytic infiltrate, he will maintain LV dysfunction during follow-up (included in the main analysis). LM: Lymphocytic Myocarditis; LV: Left Ventricular. A) HE showing diffuse inflammatory infiltrates and myocardial necrosis. B)Immunohistochemistry showing diffuse CD8+ T cells infiltrates (in red). C) HE showing moderate inflammatory infiltrates and mild myocardial necrosis. D) Immunohistochemistry showing diffuse HLA-DR+ cells (in red). E) HE showing poor inflammatory infiltrates and myocardial necrosis. F) Immunohistochemistry showing mild HLA-DR+ cells (in brown).

After 10 months (interquartile range, 8 to 12) of follow up, 27 patients (56%) showed a persistent LV systolic dysfunction. One of these patients underwent HTx for refractory HF at 7 months from the diagnosis (this patient was included in the main analysis since his LVEF was severely impaired at last available echocardiographic evaluation of the native heart). At baseline, signs and symptoms of HF were less severe (lower heart rate, higher systolic blood pressure, lower rate of NYHA IV class, lower frequency of fulminant form) in patients with persistent LV systolic dysfunction at follow up compared to the others. Moreover, patients with persistent LV dysfunction had a longer median duration of symptoms at admission (20 vs. 5 days, p<0.001) and, consistently, presented with a larger LV size, showed more frequently a poor (rather than plentiful) lymphocytic infiltrate at EMB, and a lower LVEF at discharge in comparison with patients without persistent LV dysfunction. Finally, they were younger and more frequently presented signs of acute inflammation (higher CRP values and pericardial effusion, Table 1, 2^nd^ and 3^rd^ columns; Fig 2). The two groups did not differ regarding the use of immunosuppressive therapy, ACE inhibitors and aldosterone receptor antagonists. Patients with the recovery of LVEF were less treated with beta-blockers with respect to the patients with persistent LV dysfunction (62 vs. 93% respectively, p = 0.012).

**Fig 3:**
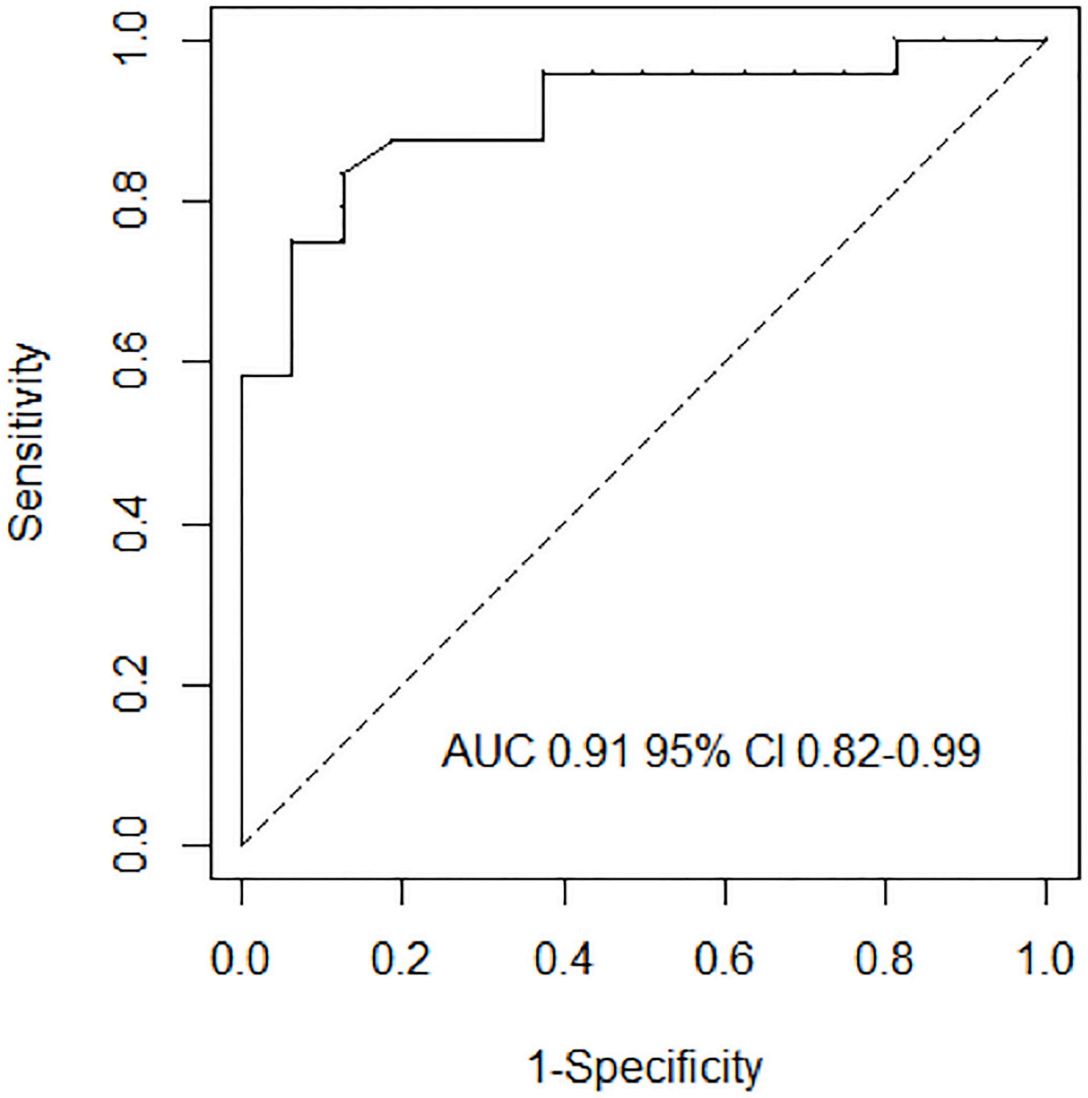
Receiver operating curves analysis for baseline prediction model of persistent LV systolic dysfunction during follow-up. The model including poor lymphocytic infiltrate + left ventricular end-diastolic diameter + non-fulminant myocarditis when surviving to the acute phase showed the highest accuracy. Legend. AUC, area under the ROC curve; CI, confidence interval; LVEF: left ventricular ejection fraction;

Table 2 shows the baseline predictors of persistent LV systolic dysfunction at univariable (left panel) and multivariable (right panel) analyses. The independent predictors of persistent LV systolic dysfunction were: higher baseline LVEDD [Odds ratio (OR) 1.22, 95% confidence interval (CI) 1.04–1.43, p = 0.002], non-fulminant presentation [OR 8.46, 95% CI 1.28–55.75, p = 0.013], and a poor lymphocytic infiltrate [OR 12.40, 95% CI 1.23–124.97, p = 0.010]. ROC analysis confirmed the highest accuracy of the model based on these variables compared to the other possible models (area under the curve [AUC] 0.91, 95% CI 0.82–0.99, Fig 3, and S1 Table). Internal validation of this model showed a maximum error in predicted probabilities of 0.08, and a bias-corrected AUC of 0.898.

**Table 2:** Univariable and multivariable analyses for persistent LV systolic dysfunction.

	Univariable			Multivariable		
	OR	95% C.I.	p	OR	95% C.I.	p
Age^a^	1.06	1.01–1.11	0.015			
Heart Rate^a^	0.96	0.93–0.98	0.001			
SBP^a^	1.10	1.020–1.192	0.014			
**Non-fulminant forms (if surviving to the acute phase)**	**14.87**	**3.60–61.39**	**<0.001**	**8.46**	**1.28–55.75**	**0.013**
**Poor Lymphocytic Infiltrate**	**9.45**	**1.77–50.47**	**0.009**	**12.4**	**1.23–124.97**	**0.010**
**Baseline LVEDD**^a^	**1.19**	**1.06–1.34**	**0.003**	**1.22**	**1.04–1.43**	**0.002**
Baseline LVEDV^a^	1.02	1.00–1.04	0.018			
Pericardial Effusion	0.18	0.04–0.80	0.024			
Baseline increased CRP	0.089	0.02–0.39	0.001			

SBP, systolic blood pressure; EF, ejection fraction; LVEDD, left ventricular end-diastolic diameter; LVEDV, left ventricular end-diastolic volume; MR, mitral regurgitation; RFP, restrictive filling pattern; TAPSE, tricuspid annular plane systolic excursion; IABP, intra-aortic balloon pump; ECMO, extracorporeal membrane oxygenation; CRP, C-reactive protein; EMB, endomyocardial biopsy;

^a^. Odds ratio estimation is referred to every unit increase for continuous variables.

#### LVEF at discharge and persistent LV dysfunction at follow-up

LVEF measured at pre-discharge echocardiogram was significantly lower in patients that later showed persistent LV dysfunction at 1-year follow-up compared to the others (32%±8 vs. 53%±8 respectively, p<0.001). At discharge, LVEF was already > 50% in the majority of the patients that showed normal LV systolic function at 1-year follow-up (17/21, 81%). On the contrary, only one out of the 27 patients with persistent LV dysfunction at 1 year (3.7%) exhibited a normal LVEF of 55% at discharge, which deteriorated to 35% at 12 months.

LVEF measured at pre-discharge had a similar accuracy in predicting persistent LV dysfunction at follow up compared to the admission model (AUC 0.946 95% CI 0.882–1.00).

### Survived fulminant forms

Twenty-three (48%) patients of our population had fulminant acute myocarditis survived after the acute phase. They were treated with intravenous inotropes, plus mechanical support in 13 (57%) of the cases. Among them, 83% had at least a moderate lymphocytic infiltrate and the majority of them (78%) received immunosuppressive treatment. Six out of the 23 fulminant forms (26%) showed persistent LV dysfunction at follow up. Compared with patients with fulminant presentation who recovered, they had similar baseline LVEF (22%±3 vs. 22%±9, p = 0.96) but a less frequent plentiful infiltrate at EMB (6 vs. 50%, p = 0.03) and a lower rate of normalized LVEF at pre-discharge (0 vs. 65%, p<0.001). (S2 Table).

### Long-term outcome

**Fig 4:**
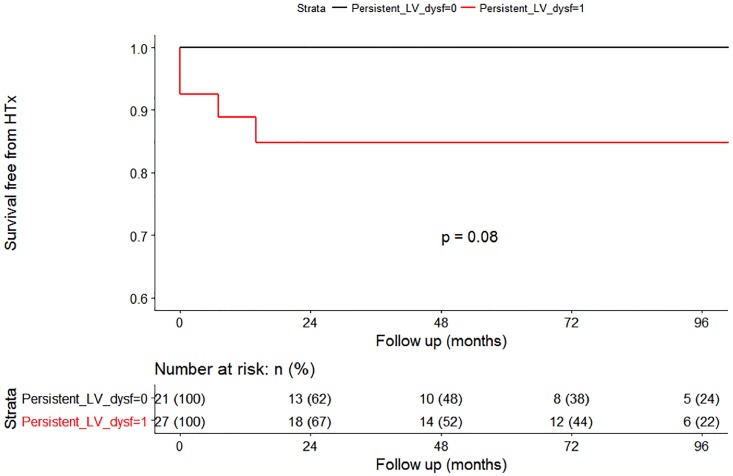
Central illustration. Long-term D/HTx-free survival curves according to the persistent LV systolic dysfunction during follow-up. The curves start from follow-up revaluation. Legend. LV: Left Ventricular; D/HTx: Death or Heart Transplantation.

During a median follow-up of 48 (interquartile range 16–94) months, patients with persistent LV dysfunction at follow-up showed a trend for higher rates of cardiac death or HTx compared with patients showing normalized LVEF (p = 0.08, Fig 4). In the subgroup characterized by recovery of LVEF during follow-up, there were 4 events (all occurred within the first 30 months of follow-up). Conversely, no events in the subgroup characterized by of LVEF during follow-up.

## Discussion

This study evaluates a cohort of patients with acute LM, presenting with HF symptoms of recent onset (≤ 30 days) and LV dysfunction (mean LVEF at admission 26%). To the best of our knowledge, this study is the first report on persistent LV systolic dysfunction in histologically proven lymphocytic myocarditis, and provides some important insights: 1) more than half of the patients surviving the index hospitalization exhibited persistent LV systolic dysfunction, at discharge and during follow-up; 2) fulminant forms were characterized by a poor outcome during hospitalization confirming recent studies [8] 3) increased LV size, poor lymphocytic infiltrate at EMB, non-fulminant presentation at admission, and lack of improvement of LVEF at pre-discharge echocardiography predicted persistent LV systolic dysfunction at follow-up in survivors.

### Frequency and predictors of persistent LV dysfunction at follow up

Although LV systolic dysfunction is not very common in patients presenting with acute LM, this setting constitutes a diagnostic and management challenge. [5] These patients often present viral prodromal symptoms, but the causative agent remains frequently unknown despite the use of real time PCR techniques aimed at detecting the specific viral agent in the myocardium[17]. Our study shows that at 1-year follow-up, LVEF is <50% in more than half of patients with LM presenting with LV systolic dysfunction at admission. Intriguingly, features of a sub-acute inflammatory disease already present at the clinical onset, suggesting an evolving progressive cardiomyopathy process (i.e. poor lymphocytic infiltrate at EMB, dilated LV, and non-fulminant presentation), appear to be related to persistent LV dysfunction at follow-up. Despite a maximum of 30 days of symptoms as inclusion criterion of this study, it is possible that patients with persistent LV dysfunction had been evaluated and treated later along the course of the inflammatory process in comparison with patients who fully recovered. In fact, a younger age as well as an increased C-reactive protein and pericardial effusion at admission were more frequent in patients who recovered compared with those who did not. While inflammation tend to spontaneous resolution over time, patients that did not recover showed only a poor inflammatory infiltrate as probably a relevant quota of damage already occurred with lower probability of LVEF recovery despite the implementation of optimal therapeutic strategies (including early supportive therapy, standard HF therapy, immunosuppression). Finally, patients with persistent LV systolic dysfunction showed a trend of worse long-term prognosis compared to patients that normalized LVEF. Interestingly, the overall event rate was relatively low, and all the events occurred in the first 3 years after diagnosis confirming recent reports on post-myocarditis dilated cardiomyopathy [18].

### Fulminant forms

Fulminant forms comprised almost half of our cohort. It must be noted that more than 80% of fulminant cases who survived presented at least moderate inflammatory infiltration and most of them were treated with steroids. More than 75% of patients with fulminant myocarditis who survived after the acute phase showed a normal LV systolic function already at the discharge. This could explain the apparently paradoxical low percentage of beta-blockers in the group of patients with LVEF recovery at follow-up evaluation. However, 26% of survived fulminant myocarditis were characterized by persistent LV systolic dysfunction at 1 year. Notably, they were characterized by a poor inflammation at EMB performed at index hospitalization. These finding underscores the need of individualized long-term follow-up and therapy [8].

### Clinical implications

This study considers one of the largest cohorts of biopsy-proven acute LM patients with LV systolic dysfunction at presentation, and some clinical implications for patient management and further studies may be derived: 1) early endomyocardial biopsy is highly recommended in patients with clinically suspected myocarditis and newly diagnosed significant LV dysfunction: these patients should be promptly referred to experienced centers for both diagnosis and treatment, that may include advanced circulatory support and transplantation; 2) time course of LV dysfunction must be followed and characterized during index hospitalization, since significant improvement of LVEF may occur, and pre-discharge LVEF predicts long-term LV function; 3) fulminant myocarditis portends a high risk for early death or need for HTX, but full recovery is possible, often persistent over time. This observation, together with the high rate of persistent LV dysfunction and its association with dilated LV and smoldering inflammation at EMB at admission -which may reflect delayed diagnosis- and some recent evidences [19], suggest to consider a prospective study on early steroid therapy in patients with acute lymphocytic myocarditis and severe LV dysfunction.

### Limitations

This study suffers from the intrinsic methodological limitations of all observational retrospective studies. Moreover, the study population derives from tertiary referral centers and this constitutes a selection bias. Our study considered a small number of patients and events and this explain wide confidence intervals of the estimates, even if the boostrap-based internal validation of the regression model gave satisfactory results.

Troponin essays used in the enrolling Centers were different. As a consequence, it was not possible to use the troponin values at diagnosis as included variable, despite it was generally increased in the study patients.

Data derived from cardiac magnetic resonance (CMR) or laboratory data such as natriuretic peptides and troponin were available in a minority of our patients and therefore were not considered. This limitation is due to the long enrollment period with patients enrolled more than 10 years ago, when especially CMR use was not as widespread as today. The relevance of late gadolinium enhancement detected at CMR can provide further hints to identify patients at risk to maintain LV dysfunction at follow up evaluation as suggested by recent studies [6].

Given the retrospective and observational nature of the study and the differences in timing of initiation, drug combination and doses, and duration of immunosuppressive therapy, no inferences can be derived about the role of immunosuppression in acute myocarditis with LV dysfunction.

## Conclusions

Patients with acute lymphocytic myocarditis presenting with LV systolic dysfunction exhibit a persistent LV impairment at 1-year in more than 50% of the cases.

Features suggestive of a poor inflammatory process associated with cardiac enlargement, and lack of early recovery, emerged as potential useful tools for predicting persistent LV systolic dysfunction during follow-up.

## Supporting information

S1 Table Performance of the principal different multivariable model at admission. (DOCX)

S2 Table Patients with survived fulminant forms: Baseline characteristics of patients with versus without persistent left ventricular systolic dysfunction. (DOCX)
