# Supplementary material for: Persistent left ventricular dysfunction after acute lymphocytic myocarditis: Frequency and predictors
Source: PLoS One. 2019 Mar 28;14(3):e0214616. doi: 10.1371/journal.pone.0214616 (PMC6438511; doi:10.1371/journal.pone.0214616)
Supplement: S1 Table — (DOCX) [file pone.0214616.s001.docx]

**Supplementary table 1: performance of the principal different multivariable models at admission**

|  | **ROC Curve** | | |
| --- | --- | --- | --- |
|  | **AUC** | **95% C.I.** | **P** |
| **Model 1 (Non-fulminant forms surviving to the acute phase+ Poor Lymphocytic Infiltrate + Baseline LVEDD)** | **0.909** | **0.815-1.000** | **<0.001** |
| Model 2 (Non-fulminant forms surviving to the acute phase + Poor Lymphocytic Infiltrate + Baseline increased CRP) | 0.891 | 0.792-0.991 | <0.001 |
| Model 3 (Non-fulminant forms surviving to the acute phase+ Poor Lymphocytic Infiltrate + Baseline LVEDV) | 0.885 | 0.774-0.995 | <0.001 |
| Model 4 (Heart Rate + Poor Lymphocytic Infiltrate + Pericardial Effusion | 0.879 | 0.766-0.993 | <0.001 |
| Model 5 (Non-fulminant forms surviving to the acute phase + Baseline LVEDD) | 0.864 | 0.804-1.000 | <0.001 |
| Model 6 (Poor Lymphocytic Infiltrate + Baseline LVEDD) | 0.858 | 0.737-0.979 | <0.001 |
| Model 7 (Non-fulminant forms surviving to the acute phase + Poor Lymphocytic Infiltrate) | 0.833 | 0.705-0.962 | <0.001 |
| Model 8 (Non-fulminant forms surviving to the acute phase + Poor Lymphocytic Infiltrate+ST-supraelevation) | 0.806 | 0.668-0.944 | 0.002 |

LVEDD, left ventricular end-diastolic diameter; LVEDV, left ventricular end-diastolic volume;

CRP, C-reactive protein.
