# Supplementary material for: Persistent left ventricular dysfunction after acute lymphocytic myocarditis: Frequency and predictors
Source: PLoS One. 2019 Mar 28;14(3):e0214616. doi: 10.1371/journal.pone.0214616 (PMC6438511; doi:10.1371/journal.pone.0214616)
Supplement: S2 Table — (DOCX) [file pone.0214616.s002.docx]

**Supplementary table 2: Patients with survived fulminant forms: Baseline characteristics of patients with versus without persistent left ventricular systolic dysfunction**

|  | **Fulminant forms**  **surviving to the acute phase**  **(N=23)** | **Fulminant forms**  **with persistent LV systolic dysfunction**  **(N=6, 26%)** | **Fulminant forms without**  **persistent LV systolic dysfunction**  **(N=17, 74%)** | **P** |
| --- | --- | --- | --- | --- |
| **Baseline LVEDD (mm)** | 54±10 | 58±5 | 52±11 | 0.177 |
| **Baseline LVEF (%)** | 22±8 | 22±3 | 22±9 | 0.956 |
| **LVEF at discharge (%)** | 47±14 | 29±5 | 54±9 | <0.001 |
| **LVEF at discharge** ≥ **50%, n (%)** | 15 (65) | 0 | 15 (65) | <0.001 |
| **Poor (vs. moderate to plentiful) lymphocytic infiltrate, n (%)** | 4 (17) | 3 (50) | 1 (6) | 0.03 |
| **Immunosuppressive therapy n (%)** | 18 (78) | 5 (83) | 13 (76) | 0.726 |

LVEDD, left ventricular end-diastolic diameter; LVEF, ejection fraction.
